# Chocolate Ganaches: Formulation, Processing and Stability in View of the New Production Trends

**DOI:** 10.3390/foods13162543

**Published:** 2024-08-15

**Authors:** Barbora Lapčíková, Lubomír Lapčík, Tomáš Valenta, Vojtěch Neuwirth

**Affiliations:** 1 Department of Food Technology, Faculty of Technology, Tomas Bata University in Zlin, Nám. T.G. Masaryka 5555, CZ-760 01 Zlin, Czech Republic; lapcikova@utb.cztvalenta@utb.cz (T.V.); v_neuwirth@utb.cz (V.N.); 2 Department of Physical Chemistry, Faculty of Science, Palacky University Olomouc, 17. Listopadu 12, CZ-771 46 Olomouc, Czech Republic

**Keywords:** ganache, pralines, recipe formulation, ganache processing, chocolate, emulsion, oleogels, hydrocolloids

## Abstract

This review aims at the current trends in chocolate ganache production and recipe formulation. Ganache is a blend of chocolate, sugars, dairy, and other ingredients commonly used to fill pralines, pastries, etc. In spite of ganache’s popularity in the food industry, a comprehensive review focused on the application of functional substances and ganache processing has not been discussed in the scientific literature. This review addresses the new ways of applying special ingredients, such as vegetable fats and seeds, flavor infusions, oleogels, hemp products, etc., which can be added to the ganache matrix to achieve desirable properties. In particular, the application of sterols and sterol esters as functional substances of oleogels seems to be a very promising method, enhancing the ganache fat profile. The elevated caloric content that is characteristic of ganache can be substantially attenuated through the application of hydrocolloids and/or fruit-based components, thereby offering the potential for caloric reduction without compromising on taste. The various alterations to ganache formulations by the application of natural substances offer a large base for the development of novel ganache variants and relevant food products.

## 1. Introduction

Ganache is a globally popular confectionary product that has been manufactured for over 150 years [1]. From a food technology perspective, ganache may be defined as a mixture of chocolate, sugars, dairy components (particularly cream), fruit puree, alcohol, etc. It is utilized as a culinary item in a variety of applications, including, but not limited to, fillings in confectionary products, bakery goods, pastries, and others [2]. Some trendy foods, such as macarons (i.e., cookies prepared from sweet meringue and ground almonds), can contain a ganache. Macarons can be filled with a ganache and marmalade between two cookies, as proposed by pastry chef Pierre Hermé [3], providing the product an improved sensory complexity [4]. Another possibility is the filling of macaron shells with chocolate ganache flavored by coffee extract [5].

The compositions and preparation methods of a ganache significantly affect its functional properties [6,7]. The ganache texture, which can range from fluid to paste-like, is contingent upon the type of chocolate and specific proportions and ratios of ingredients used [8]. The method of emulsification, which involves dispersing fat droplets from the chocolate and dairy components into a water-based phase, plays a crucial role in determining the ganache texture and consistency. A well-emulsified ganache, e.g., using an emulsification time of 3 min [7], exhibits a smooth and creamy texture that is highly desirable in confectionery applications [9]. Ganache’s antioxidant activity as a health-promoting attribute can be successfully enhanced by the application of specific functional substances. In this context, Park et al. [10] observed that the addition of a powder from *Porphyra Tenera* (Nori seaweed) in a chocolate ganache resulted in an increase in DPPH (1,1-diphenyl-2-picrylhydrazyl) and SOD (*superoxide dismutase*) activity, showing an increasing trend with greater quantities of the powder (applied in the range from 1 to 3 wt.%). This higher antioxidant ability was also accompanied by an elevated hardness of the modified ganache compared to a reference product without the powder [10]. Similar applications offer new perspectives on how to produce high-quality ganache-based products with improved and tuneable characteristics.

Chocolate ganache is currently mostly used as a filling in pralines, truffles, and filled chocolates and is piped for decorations and whipped as a topping. In particular, filled bonbons of various flavors are the most favoured and desirable products [11,12,13]. Edible coatings forming a ganache layer of chocolate [14] can be adjusted in versatile products. These practical applications are based on characteristic formulations of ganache fillings. In addition to the traditional ingredients (chocolate and cream), various substances can be utilized to modify the ganache properties [6]. Health-promoting attributes of bioactive substances in specific food ingredients can be combined with the typical functional profile of a ganache mixture. For instance, as reported by Doğan and Yilmaz [12], chestnut honey rich in phenolic substances with antioxidant and antibacterial activity could be added to a ganache mixture processed from ivory drop chocolate, cream, and butter. The authors reported that the bitter taste of the chestnut honey (applied in a concentration range from 5 to 9 wt.%) was able to provide a flavor harmony when combined with the chocolate. This is an example of the successful application of “food pairing”, a new trend which is particularly popular in gastronomy. This pairing is based on the concept of flavor compatibility between various recipe ingredients, resulting in a preferable balanced flavor [12].

Despite its widespread use in the food industry, the scientific discussion of ganache formulations and processing in relation to ganache’s functional profile remains limited. This review deals with the issue of ganache production with respect to the application of natural substances in modified recipe formulations and altered processing procedures. The new trends of ganache preparation techniques are discussed with regard to product quality and shelf-life stability. Based on the relevant scientific databases (ScienceDirect, Wiley Online Library, Taylor & Francis database, and Google Scholar), the keywords “chocolate ganache”, “chocolate filling”, “praline filling”, and “ganache filling” were employed for searching for and selecting scientific articles, theses, and other up-to-date manuscripts to complete this review. The keywords indicated tens to maximally hundreds of articles published in peer-reviewed journals, showing a relatively limited number of scientific references. To the best of our knowledge, no similar review summarizing the subject of our research has been presented at the time of this review’s compilation.

Future physico-chemical and sensorial research should focus on exploring the effects of the application of various ingredients and processing procedures on ganache’s functional attributes. In particular, scientific studies can investigate the use of alternative ingredients for creating novel ganache variants with improved nutritional profiles and unique sensory characteristics.

## 2. Ganache Nature

Ganaches are classified as emulsions, which are constituted by droplets of one phase being finely dispersed within another phase. Hence, ganache emulsions are metastable foodstuffs with kinetic stability predominantly ensured by amphiphilic molecules, or emulsifiers, adsorbed at the oil/water interface, such as surfactants or proteins. The type of emulsion, whether oil-in-water (O/W) or water-in-oil (W/O), is dependent on several factors, including the manufacturing process, the choice of emulsifiers, and the ratio of the two immiscible phases. The continuous phase of the preferred emulsion is the one that solubilizes the emulsifier. Proteins such as casein, naturally present in the aqueous phase of a heavy cream, facilitate the formation of O/W ganaches. On the other hand, oil-soluble emulsifiers like lecithin or polyglycerol polyricinoleate (PGPR) tend to form W/O ganaches [8,15,16].

**Figure 1:**
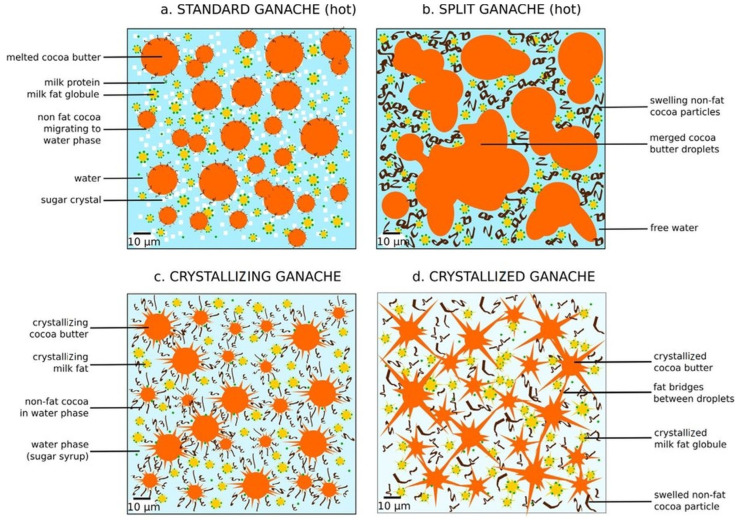
Diagrams of ganache emulsion in different forms with specific partition of substances after emulsification, as illustrated by parts (**a**–**d**): (**a**) hot standard ganache a few minutes after emulsification; (**b**) split ganache a few minutes after emulsification; (**c**) standard ganache during crystallization several hours after emulsification (stored at 16 °C); (**d**) standard ganache after crystallization when stored for several days at 16 °C. Reproduced with permission from Ref. [18]. Copyright 2018 Elsevier.

The characteristics of ganache emulsions are the subject of scientific debate. A ganache may be defined as an oil-in-water (O/W) emulsion type, formed by adding cream to chocolate. Within this mixture, cream is regarded as the aqueous phase, while chocolate constitutes the fat phase, encompassing cocoa particles that absorb water, thereby thickening the aqueous phase [17,18]. The fat suspension (chocolate) and the oil-in-water emulsion (cream) are inherently immiscible. Upon mixing chocolate and cream, the sugar from both components dissolves in water, resulting in an emulsion structure where droplets of one phase are finely dispersed within another [19]. The occurrence of partial coalescence among fat droplets can give rise to a fat network. This phenomenon can culminate in a formation of a water-in-oil (W/O) ganache emulsion [20], or a bicontinuous system, where the water and fat networks are intertwined [21]. A standard hot ganache is an O/W emulsion, where milk fat droplets are stabilized by milk proteins and non-fat cocoa particles tend to migrate from the cocoa butter to the water phase, as illustrated in Figure 1a. When cooling, the cocoa butter and milk fat begin to crystallize (Figure 1c). After crystallization, the fat crystals form bridges between the fat droplets (Figure 1d) relating to the partial coalescence, as mentioned above. In this state, non-fat cocoa particles swell with the free water remaining in the system, thereby contributing to the stabilization of the ganache structure. In contrast to a standard ganache, the structure of a split ganache is unstable and heterogeneous, not being classified as an emulsion. In a split ganache, a high number of fat droplets leads to their merging before crystallization can occur, as can be seen in Figure 1b. Furthermore, a split ganache has a higher concentration of non-fat cocoa particles and a lower concentration of sugar, enabling the non-fat cocoa particles to rapidly migrate to the water phase, which is characterized by a high amount of free water. This process causes the aqueous phase to thicken, complicating the process of separating the fat droplets even more [18].

## 3. Research Progress and Development of Ganache Formulation and Processing

### 3.1. Ganache Formulation

While there is not a universally accepted method for ganache manufacturing, the traditional art of ganache preparation can involve a variety of techniques. Most ganache recipes incorporate a high content of water from ingredients like cream or fruit purée, which contributes to ganache’s appealing smooth texture. The proportion of cream used in a recipe can vary, typically ranging from 9 to 41 wt.% [22]. A 2:1 ratio of chocolate to cream is optimal for dark and semi-sweet chocolate ganache; 2.5:1 or 3:1 ratios are optimal for producing ganache with milk or white chocolate [2]. In industrial conditions, hot cream is conventionally poured into molten chocolate [8]. Specifically, cream heated to 40 °C is added to liquid tempered chocolate followed by mixing. An alternative method for preparing ganache involves the process of pouring near-boiling cream over solid chocolate at room temperature. The heat from the cream facilitates the melting of the chocolate, and the mixture is then stirred until it achieves a homogeneous consistency. The cocoa butter in the chocolate, although primarily in a melted state, needs to have an adequate quantity of β-V crystals. These crystals act as seeds for the cocoa butter when it cools down to room temperature. If this condition is not acquired, the resulting ganache may have a grainy texture [2,23].

In the culinary blend of a ganache, the sugar crystals and milk powder from the chocolate partially dissolve in the cream’s water, which is rich in sugars (mainly lactose) and water-soluble proteins. Cocoa particles, along with sucrose and milk particles that are chocolate constituents, exhibit an average dimension of 10–15 μm and remain undetectable to sensory perception within the mouth [24]. These cocoa particles do not fully dissolve in the aqueous phase of the ganache due to their content of insoluble fibers and starch, but they can contribute to the stabilization of the emulsion system by migrating to the interfaces [18]. Within the emulsion phase, the nonuniform fat globules are structured by both cocoa powder particles and cocoa butter crystals [2]. Non-fat cocoa and milk proteins are dispersed in the aqueous phase of the ganache and could undergo swelling or partial solubilization. The crystallization process within the fat phase holds the potential to either disrupt or stabilize the emulsion system, facilitated by the partial coalescence of fat droplets, which, in turn, form a fat network that reinforces the ganache structure [18].

As presented by McGill and Hartel [2], the variation in ganache formulations using different solid fat contents (SFCs) and types of chocolate can result in alterations to the ganache structure, manifested by changes in the ganache texture, rheology, and melting profile. The authors found that the texture of ganache can be controlled by adjusting the proportion of defatted cocoa powder (relative to sugar) or non-fat milk powder when using a chocolate system with a constant fat content. Increasing the SFC of a ganache emulsion (i.e., elevating the levels of cocoa particles) can provide a ganache with a harder and less spreadable texture that is more resistant to deformation. Therefore, ganache created with chocolate containing higher levels of cocoa particles, particularly bittersweet chocolate, may yield a firmer structure. The ganache structural characteristics are not only influenced by the content of chocolate crystalline fat but also by the volume of the particle phases.

**Figure 2:**
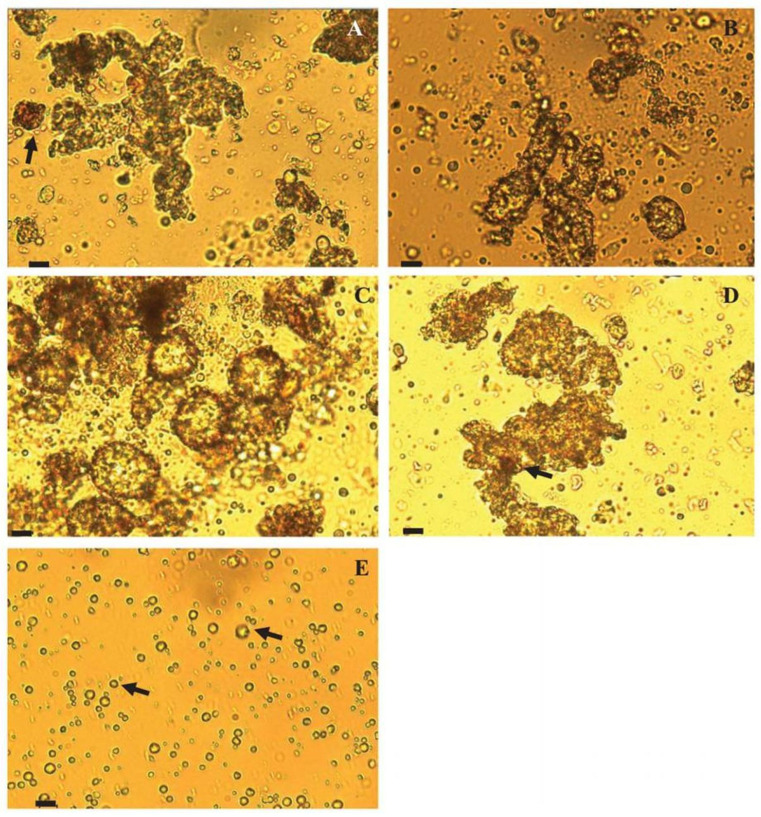
Light microscopy images showing the effect of different formulations on ganache microstructure. The diluted ganache formulations were prepared with different ratios of milk fat from cream (intact milk fat globules) and from butter (fragmented milk fat globules). The figure parts A-D depict formulations with the following weight ratios of milk fat from cream to butter: (**A**) 0:1, (**B**) 3:7, (**C**) 3:2, and (**D**) 1:0. Part (**E**) shows the cream diluted with water only. Scale bars represent 10 µm size at 400× magnification; arrows in parts (**A**) and (**D**) indicate cocoa powder in ganache formulations, and arrows in part (**E**) indicate milk fat globules in the cream. Reproduced with permission from Ref. [2]. Copyright 2018 John Wiley and Sons.

As can be seen in Figure 2, ganache samples investigated by McGill and Hartel were characterized by fat globules of various sizes and shapes, with no significant differences between the samples prepared from different dairy sources (cream or butter). The sugar present in the ganaches appeared to be dissolved without any visible crystals being detected. The cocoa powder particles (shown in a reddish-brown color in Figure 2) were distributed in two ways: some of them remained within the fat globules, while others moved into the aqueous phase. This phenomenon could be related to the hydrophobic or hydrophilic nature of cocoa powder substances. Nonetheless, there were no clear patterns observed in the distribution of cocoa particles among the different phases. Intact milk fat globules were observed in the diluted cream (as shown in Figure 2E), but these structures were not more common in the ganaches with a higher cream content. In addition, these globules could also be found in the ganache prepared from butter (Figure 2A). Based on these facts, it can be concluded that cream interacts with cocoa butter and is likely present within the primary irregular globular structures [2].

### 3.2. Future Trends in Ganache Formulation

In addition to the primary ingredients, various substances can be incorporated into the ganache recipe to achieve the desired properties of commercial products. Various new trends can be employed in the production of confections, pralines, and ganache-filled products. In particular, the high caloric content that is characteristic of ganaches can be effectively moderated through the application of food hydrocolloids, plant-based ingredients, vegetable fats, and low-calorie supplements [8,25]. For instance, reduced-fat chocolate fillings can be produced applying iota-carrageenan, being characterized by similar properties compared to the control filling [26]. In addition to this, the substitution of cream and other high-calorie components can be attractive for vegans, lactose-intolerant consumers, and consumers on a diet who appreciate reformulated chocolate products with vegetable- and other plant-based ingredients [27].

#### 3.2.1. Ganache with Vegetable Cream and Coconut Milk

To favorably change the calorie profile of ganaches, Kim et al. [28] investigated various chocolate ganache types composed of milk, vegetable cream, or coconut milk as whipped cream substitutions. The chocolate ganache made from whipped cream (with a ratio of dark chocolate and cream of 1:1) was designated as a control group. The utilized ingredients affected parameters such as the ganache moisture, crude fat, sugar content, and hardness. The whipped cream chocolate ganache, characterized by the lowest moisture (22.6 wt.%) and highest fat content (16.8 wt.%), demonstrated a median hardness (around 30 kg, i.e., 294.3 N). The milk chocolate ganache, possessing the highest moisture (37.9 wt.%) and lowest fat (6.7 wt.%) content, mirrored the results of the whipped cream sample. The vegetable cream also exhibited results similar to that of the whipped cream, with the exception of the hardness being two times higher for the vegetable cream ganache. The samples with vegetable cream and coconut milk yielded comparable product circumferences, whereas the height of the vegetable cream was significantly greater compared to the coconut milk variant. This can be attributed to the higher concentration of fat, protein, and sugar in vegetable cream in contrast to the coconut milk sample with a higher water content [28].

The ganache containing coconut milk displayed the softest texture and was sensorially evaluated as the smoothest, followed by the variants containing vegetable cream, whipped cream, and milk. In terms of the overall evaluation, the vegetable cream ganache was found to be the highest rated, followed by the whipped cream, milk, and coconut milk samples. The vegetable cream variant gained favor across all the sensory parameters tested (sweetness, hardness, texture, and overall acceptance) and was rated as the sweetest, while the coconut milk variant had the highest rating for smoothness. The differences in the sensory attributes of the ganaches can be related to their diverse nutritional profiles. As shown in Table 1, the vegetable cream and coconut milk ganache variants had lower calorie and fat contents compared to the traditional whipped cream chocolate ganache. Owing to the favorable nutritional profiles and high smoothness of alternative ganaches, vegetable oils/fats and coconut milk may be superior ingredients for the production of chocolate ganaches. Notwithstanding the less sweet sensory profile determined for the coconut milk ganache variant, the use of coconut milk in combination with alternative low-calorie sweeteners (e.g., tagatose) could be utilized in classical as well as vegetarian cuisines. Coconut milk ganache can also be used to produce high-quality chocolate snacks and could thus potentially be more beneficial to consumers’ health [28].

#### 3.2.2. Ganache with Fruit Addition

A high potential for the addition of fruit ingredients, particularly fruit puree, offers new perspectives on ganache formulation. As investigated by Neuwirth et al. [7], raspberry puree can be effectively used as a substitute for cream in the ganache mixture during praline production. In the recipe formulation, the authors used a 32.23 wt.% of puree (composed of 100 wt.% frozen raspberries) to formulate ganache with a high fruit content ratio, as shown in Table 2. Crystal sugar, glucose, fructose, and sorbitol were dissolved in the puree, which was subsequently added to a butter–chocolate blend and mixed together [7]. Moreover, a stepwise addition of raspberry puree in the ganache mixture during the emulsification process was studied (80 wt.% of the puree was emulsified in the mixture for 1.5 min, and then the remaining 20 wt.% was added and emulsified for 1.5 min). This method produced a ganache with a less elastic structure characterized by a lower firmness and cohesiveness. This indicated that the applied procedure can be used to manufacture softer and less elastic products. In accordance with rheological behavior reported for chocolates [29,30], chocolate masses [31], and chocolate pastes [32], the ganaches showed a pseudoplastic behavior pattern, characterized by a decrease in the dynamic viscosity with the increase in the shear rate. The ganaches under study had an essentially viscoelastic nature, as indicated by the phase angle *tan δ <* 1, where the elastic storage modulus prevailed over the viscous loss modulus in the entire frequency range (0.1–20 Hz) [7].

In terms of the calorie content, the energy value of the basic ganache with raspberry puree was 371 kcal (1560 kJ). From a nutritional point of view, this value represents a favorable result, indicating that the energy level of the ganache was reduced by 42% compared to the ganache of the same recipe formulation but substituting the puree with ghee (anhydrous milk fat) [33].

#### 3.2.3. Ganache with Application of Hydrocolloids

Following the application of mechanical shear to form a ganache, the emulsion system requires stabilization. This can be achieved by the application of hydrocolloids to avoid instabilities, including, but not limited to, Ostwald ripening, phase separation, coalescence, and aggregation, whereby large oil globules form due to the uptake of small oil molecules in the aqueous phase [34].

Izzreen et al. [1] investigated the addition of carrageenan, pectin, and xanthan gum at varying concentrations (0.1, 0.3, and 0.5 wt.%) to white chocolate ganache, and the effects on the ganache chemical properties (pH, moisture content, water activity, and total soluble solids), emulsion stability, viscosity (including consistency and flow behavior index), and particle size distribution were studied. The formulations of the ganaches with hydrocolloids under study are presented in Table 3. Adding hydrocolloids did not affect the pH and total soluble solid content of the ganache samples, yet, depending on the concentration, it led to decreased moisture contents and water activity values, resulting in improved emulsion stability (98–99%). The ganache samples also became significantly more viscous and less Newtonian when hydrocolloids were applied, and carrageenan and pectin at low concentrations produced a desirable majority particle size of less than 30 µm. Xanthan gum at all concentrations led to a larger particle size of the ganache samples, ranging between 70 and 115.0 µm. In spite of the highest significant increase (*p* ˂ 0.05) in the ganache viscosity after the addition of xanthan gum, the xanthan gum was found to induce the flocculation of oil droplets, resulting in the formation of a three-dimensional network structure through the self-entanglement of the xanthan gum, and in the fast creaming, as previously observed by Galanakis [35]. Due to the fact that pectin at higher concentrations in an emulsion system is also prone to depletion flocculation [36], carrageenan may be considered as a more appropriate hydrocolloid for enhancing ganache viscosity. Therefore, Izzreen et al. [1] recommend the addition of low-concentration carrageenan (0.1 wt.%) as the most effective method to improve the emulsion stability, viscosity, and particle size distribution of white chocolate ganaches. The water activity (*a_w_*) values analyzed in the samples ranged between 0.70 and 0.74, which were lower than those reported by Dias et al. [37], who observed a higher *a_w_* level exceeding 0.80 in a chocolate filling. This water activity led to the formation of cracks in the filling. The cracking phenomenon resulted in moisture loss during storage due to the interaction between soluble solids and water. This suggests that chocolate ganache, when combined with various hydrocolloids, as studied by Izzreen et al. [1], can inhibit the migration of water and fat whilst preventing microbial growth. Therefore, it may be inferred that hydrocolloids (contingent upon their concentration) maintain the water content during ganache storage and significantly influence the ganache *a_w_* level.

Dias et al. [26] evaluated the effect of iota-carrageenan on improving the shelf-life of reduced-fat white chocolate fillings. In this study, two batches of ganache fillings composed of white chocolate, skimmed milk, sugar, and either 0.5 wt.% or 1.0 wt.% of iota-carrageenan, respectively, were produced along with a control filling (as shown in Table 3) and stored for 12 months at 4 °C. The results showed that the iota-carrageenan positively influenced the rheological properties and moisture retention of the fillings; the moisture content, *a_w_*, and pH levels decreased over time, while all the formulations presented a shear-thinning behavior (flow behavior index *n* < 1) that was correlated with the iota-carrageenan concentration and storage time. Both carrageenan concentrations showed a positive correlation with the storage time through digital image analysis, although the percentage content of iota-carrageenan in the filling had no significant effect. Despite the fact that the use of hydrocolloids may increase the contamination risk if no additional heat treatment is adopted, the total counts of microorganisms (CFU) obtained at the end of the storage time (below 2.5 × 10^2^ CFU/g) were acceptable for all the formulations. The study findings showed that the use of iota-carrageenan enabled the production of reduced-fat chocolate fillings that were comparable to the standard formulation, and they retained the bulk of their original properties and adhered to food safety standards under storage conditions of 4 °C [26].

#### 3.2.4. Ganache with Chilli Flavor

The study by Seçuk and Seçím [38] was designed with the objective of formulating a ganache filling as a key component in filled chocolates with a chilli flavor. In the initial phase, the various chilli-infused ganache fillings were composed of white chocolate drops (with a minimum cocoa butter ratio of 29.5 wt.% and a minimum milk fat ratio of 6.3 wt.%), of unsalted butter with a minimum 82% milk fat ratio, whipping cream with a minimum 35% milk fat ratio, and red-hot chilli pepper or pure 100% chilli pepper seed oil. The compositions of the ganache fillings are presented in Table 4. These fillings were utilized in the manufacturing of final products, which included ganache-filled chocolates from the control group (CG), those infused with powdered chilli pepper (PCP), and those containing chilli pepper seed oil (CPS). The edibility of the chilli-ganache-filled chocolates was ascertained through sensory and physicochemical analyses. All samples underwent a 30-day storage period and were analyzed at intervals of 0, 15, and 30 days. The authors revealed a sensory preference for the CPS-ganache-filled chocolate samples over the PCP samples in terms of color and brightness (*p* < 0.05). No statistically significant differences (*p* > 0.05) in color and brightness (appearance), smell, chilli taste, and hardness were observed for the samples after storage for 0–15 days. However, the analysis by the 30th day of storage showed a significant decrease in hardness, smell, and chilli taste as well as a significant decrease in L* (brightness) and a corresponding increase in b* values (chromaticity on a blue(-)-to-yellow(+) axis). Viscosity measurements demonstrated that the CPS ganache exhibited lower viscosity compared to the control group and PCP samples [38].

#### 3.2.5. Ganache with Sacha Inchi Seeds

Plant seeds represent other source of food ingredients that can be incorporated into a ganache filling. In the study presented by Izzreen et al. [39], a ganache with 10 wt.% Sacha inchi seed paste (*Plukenetia volubilis* L.) as a substitution for whipping cream was developed (Table 5) and applied as a filling of milk and dark chocolates. The researchers found that the application of Sacha inchi could produce ganaches with an acceptable sensory profile and improved antioxidant properties. The sensory evaluation results showed that the milk and dark chocolates with the Sacha inchi ganache had an overall acceptance score (5.07 and 5.04, respectively) comparable to milk and dark chocolates without Sacha inchi (5.48 and 5.56, respectively). The addition of the Sacha inchi in the ganache formulation led to a significantly higher (*p* < 0.05) free radical scavenging activity for the milk chocolate (79.41%) and dark chocolate (90.42%) compared to the relevant samples without Sacha inchi (76.05% and 88.04%, respectively). The study also found that the application of the Sacha inchi resulted in a higher total phenolic content (4.27 mg of gallic acid/g for the milk chocolate, and 12.21 mg of gallic acid/g for the dark chocolate). The crude fiber content in both chocolates also increased (above 15 wt.%) after the addition of the Sacha inchi, showing the potential of Sacha inchi to improve the nutritional values of ganaches. According to the results of a texture analysis, both the milk and dark chocolates with and without Sacha inchi showed similar textural patterns (*p* > 0.05), with the highest hardness (13.47 N) observed for the milk chocolate with Sacha inchi [39].

#### 3.2.6. Ganache with Cannabis (Hemp) Ingredients

In the chocolate industry, cannabis needs to be added in a fat-based form in order to be miscible with chocolate. This is due to the fact that the addition of even small water amounts can cause uncontrolled hardening (chocolate seizing). This happens when water molecules interact with the sugar in melted chocolate, leading to the formation of clusters that cannot stay suspended. Chocolate subjected to this condition is nonviable for further use. However, when the water content in the chocolate exceeds approximately 20 wt.%, it becomes sufficient to dissolve the majority of the sugar, thereby facilitating the flow of the chocolate. This condition enables the creation of bonbon fillings such as ganache [40].

In this context, a cannabis tincture (either alcohol- or glycerine-based) could be utilized to integrate cannabis into chocolate products. Additionally, cannabis-infused dairy butter could be incorporated into a ganache or other filling. Nevertheless, it presents a challenge to formulate a ganache filling with an adequately low water activity to ensure an extended shelf-life when contained within a solid bar [40]. Another possible way of applying cannabis in ganache fillings is the usage of hemp seeds and hemp oils [41]. In a registered patent, Steinbach [42] presented a process to produce pralines and chocolates utilizing hemp oil, hemp meal, hemp seeds, hemp liqueur, and bio-chocolate as primary ingredients. The pralines were made by placing hemp meal in a bowl, sprinkling it with hemp liqueur, melting bio-chocolate, folding in the hemp meal mixture, and allowing it to cool. The hemp seeds were roasted with hemp oil, and the dough sheet was cut into pieces, half-coated with melted bio-chocolate, decorated with the hemp seeds, and allowed to set. This process resulted in a formation of pralines that incorporated the nutritional benefits of hemp and indulgent qualities of bio-chocolate [42]. Nonetheless, the application of hemp ingredients in ganache formulations is limited by the current legislation, which regulates the usage of hemp in food and food supplements, including natural and synthetic cannabis extracts [43].

#### 3.2.7. Ganache with Oleogels

In confectionary products, the substitution of cocoa butter and fats by oleogels seems to be highly beneficial. Oleogels, i.e., semi-solid materials with a continuous oil phase immobilized in a 3D network structure [44], are characterized by a higher oil binding capacity and higher heat resistance [45,46]. The properties of oleogels are based on their intermolecular and fiber tubular structure formed by the aggregation of self-assembled fibrils [47]. The mixing of oleogels in a phase of low water activity (e.g., a nougat phase) is able to considerably reduce the oil migration in the system of a ganache-filled product. In recent years, scientific research has been conducted on self-assembling mixtures of plant sterols and sterol esters in edible oils and emulsions. An oleogel mixture derived from a combination of β-sitosterol and γ-oryzanol appears to be one of the most promising systems for application in food matrices [45,48].

In the research published by Wendt et al. [49], an oleogel composed of β-sitosterol and γ-oryzanol was integrated into a praline filling using different quantities of sunflower-based oleogel (2.5 or 14 wt.%) and structurant in the gel (10 or 25 wt.%). The gel was either used as a part of a nougat filling (gelled nougat) or of a chocolate phase (gelled chocolate) or used as a separate layer between a chocolate and nougat filling (three-layer system). The amount of migrated oil was regularly analyzed by differential scanning calorimetry (DSC) of the praline surface after 24 weeks of storage at 10, 18, and 28 °C. The researchers observed that the inclusion of the oleogel retarded the migration of oil within the system to a varying degree. For storage at 18 °C, the samples were characterized by limited oil migration; moreover, the praline with a gelled nougat layer with a small amount of oleogel was completely free of oil migration. For storage at 28 °C, it was found that the strongest oleogel (with 25 wt.% of structurant) was the most effective in suppressing oil migration. The DSC results showed that despite the additional oil added into the system via the oleogel, the level of oil observed in the chocolate layer was reduced through the presence of the gel. The sunflower oil from the gel and hazelnut oil from the filling seemed to be completely mixed before a significant migration was detected. In particular, the three-layer system and gelled chocolate appeared to be highly promising in suppressing oil migration.

From a structural point of view, oleogel-filled products can generate the same crystal form as dark chocolate, providing a stable crystal network and preventing the chocolate from fat blooming [46]. In addition to this, the practical application of oleogels with lower levels of saturated and trans fatty acids offers the possibility to replace conventional saturated fatty acid (SAFA)-based lipids with a healthier alternative [50] by immobilizing liquid edible oils in the filling. Oleogelation, the process of converting oil rich in essential fatty acids into a semi-solid form, offers potential in creating the necessary solid structure. Notably, this conversion process maintains the nutritional value of the added oils, ensuring that their health benefits are retained in the final product [44].

### 3.3. Ganache Processing by Altered Emulsification Time and Low-Pressure Conditions

Neuwirth et al. [7] investigated the effect of applying reduced and prolonged emulsification times (1 min and 6 min, respectively) on fruit-based ganaches in comparison to a reference ganache emulsified for 3 min. The samples’ emulsification temperature was (30 ± 1) °C at the end of the mixing procedure. The authors also studied ganache processed under low-pressure conditions (with a mixer working pressure of 0 bar) using emulsification for 3 min at a lower temperature (24 ± 1) °C. As was found, the extended emulsification time (6 min) did not have a significant effect on the ganache properties in relation to the texture, water activity, viscoelasticity, structure profile, and thermal (melting) profile. Similar textural and melting patterns were also observed for the ganache processed under low-pressure conditions (G3_LP), although it showed a significantly higher viscoelasticity compared to the reference. This could be explained by the structural changes in the ganache mixture; the lower emulsification temperature used in the G3_LP processing procedure resulted in a partial coalescence of the emulsion when crystalline fat ruptured the emulsion droplets [7]. Overall, it can be concluded that the application of longer emulsification time (6 min) and low-pressure conditions do not substantially affect the ganache viscoelasticity, texture, and melting profile as the main functional attributes important in the processing of pralines and other ganache-filled products.

In the case of the ganache emulsified for 1 min, the shorter emulsification time led to the formation of an incompletely homogenized structure. This was manifested by a reduction in the ganache viscoelasticity and shear viscosity as well as by a lower ganache elasticity and springiness. This observation was correlated with a higher ganache firmness, which increased by 112% compared to the reference [7]. As is evident, an appropriate length of the emulsification time plays a vital role in terms of both production costs and the quality of the ganache product.

### 3.4. High-Pressure Processing of Ganache

In recent years, high-pressure processing (HPP) has gained popularity as a food preservation technique. It involves subjecting packaged food to pressures between 100 and 600 MPa to eliminate microorganisms and deactivate enzymes. During HPP treatment, a hydrostatic pressure is evenly transmitted to the food inside the vessel, regardless of the food size or shape, thus minimizing the processing time [37]. While lethal to microorganisms, HPP does not break covalent bonds and has minimal effects on food chemistry. It is applied at chilled or mild temperatures (<45 °C), allowing foods to be preserved with minimal impact on flavor, texture, appearance, and nutritional value. This method may also be used for high-moisture-content and liquid foods [51] such as ganache.

In the study by Panda et al. [52], the impact of high-pressure processing (HPP) on dark chocolate fillings was investigated. The fillings were subjected to different pressure levels (400 and 600 MPa) and holding times (10 and 20 min), followed by storage at 20 °C for a time of 12 months. Interestingly, the HPP treatment did not significantly affect the moisture content, water activity (*a_w_*), and pH of the ganache fillings. However, several physical properties did change during storage [52], indicating a significant effect of the storage time and temperature on the chocolate matrix [53]. The color profile of the ganaches shifted from values comparable with commercial chocolate bars [54], showing a consistent decrease in the ganache parameter L* (brightness) over time. However, the differences between the samples in terms of the parameters L*, a* (greenness to redness), and b* (blueness to yellowness) were not significant with regard to the HPP conditions (*p* > 0.01) [52]. The rheological behavior of the HPP-treated samples evolved over time. The elastic modulus at 1 Hz (*G’*_1Hz_) increased from the 2nd to the 12th month of storage, with the final values ranging between 1603 kPa and 2139 kPa. This change may be attributed to alterations in the crystallinity of the cocoa butter during storage, affecting the stability of the ganache fat network [18], which was in accordance with the harder texture observed for stale chocolate [55]. Notably, ganache fillings treated by HPP at 600 MPa for 20 min exhibited a weaker structure after 12 months of storage [52]. These findings provide new insights into the effects of HPP on the functional profile of chocolate ganache. However, they also highlight the need for the careful optimization of HPP processing parameters, particularly in relation to the weakening of the ganache structure, representing a possible disadvantage of the application of HPP.

## 4. Effects of Recipe and Processing on Ganache Quality and Stability

There are many methods to prepare ganache products, all of which may provide the desired characteristics, but standardized recipes and procedure conditions are necessary to gain a uniform flavor, consistent texture, and other functional attributes. In particular, the type of chocolate selected for ganache production can significantly influence not only the flavor profile but also the physicochemical properties of the final product. Dark chocolate, known for its higher cocoa solid content, typically results in a ganache with a firmer structure. This is attributed to the fact that the cocoa solids contribute to the overall structural integrity. Conversely, milk and white chocolates, which are characterized by their higher milk and sugar contents, tend to produce a ganache with a softer texture. The reason behind this lies in the unique properties of sugar in chocolate; when chocolate melts, the added sugar behaves like a liquid, thereby softening the structure [2,56].

Ganache is appealing due to its soft, creamy, and smooth texture, which is especially noticeable when contrasted with a harder chocolate shell, like in a truffle. This soft and creamy texture allows for a smooth release of flavor, spreading the tastes of the chocolate and additional flavorings. A traditional ganache improves the flavor nuances of the chocolate used in the ganache processing. Many chocolate manufacturers pair the chocolate’s flavor with the flavors in the ganache to create unique and tasty products. The ingredients and processing procedures applied may alter the chocolate product’s microstructure and thus moderate the ganache properties [6]. In view of this, the addition of solid particles (such as sugar, cocoa butter, and lecithin) has a significant impact on the microstructural characteristics [57,58]. Mixing vegetable fats with ganache chocolate can lead to an eutectic effect, meaning that the interactions between the fats can result in a mixture with a lower melting point than any of its individual components [59]. The resulting ganache mixture is softer and more spreadable at room temperature than the individual fats with different melting points would be on their own. In general, the eutectic effect in ganache contributes to its unique texture that readily melts at body temperature and to its flavour profile, enhancing the overall sensory characteristics of the ganache confection [6].

However, some imperfections in ganaches can occur due to the recipe formulations and technological conditions; avoiding these imperfections is essential for the industrial manufacturing of ganache-filled products on a profitable scale. The imperfections are associated with the development of fat blooms, moisture/fat migration [60], browning reactions, changes in flavor and color [37,61], etc. The most common imperfections observed in ganache and praline confections are described in Section 4.1.

Although ganache fillings have a relatively low water activity (*a_w_*), which is not favorable for the growth of most microorganisms, the spoilage of such fillings can still occur, particularly due to the proliferation of microorganisms such as osmophilic yeasts, xerophilic fungi, and bacteria that can withstand low-*a_w_* conditions [37]. The proliferation of these microorganisms can result in off flavors, slime formation [62], gas production that causes the chocolates to crack, sour fillings, or visible fungus formation between the filling and the chocolate shell [22]. Ganache quality and shelf-life stability can be improved by adding extra ingredients. These recipe ingredients may include corn syrup or honey for sweetness, various flavor enhancers, alcohol, butter [6], hydrocolloids, fruit purees [7], etc. Butter, in particular, is often used to make a ganache softer and increase its creamy texture. Alcohol and fruit purees may be added to enhance the flavor, while ingredients such as corn syrup or honey can improve the texture and extend the shelf-life stability by controlling sugar crystallization. For example, the shelf-life of fresh cream ganache usually lasts for about two to three weeks, but adding certain ingredients can significantly extend it [6]. The shelf-life of ganache and various strategies for improving ganache stability are discussed in Section 4.2.

### 4.1. Processing Imperfections of Ganache

#### 4.1.1. Crack Formation in Ganache-Filled Products

In chocolate pralines, a soft ganache filling is surrounded by a chocolate shell. This implies a possible problem when the filling begins to migrate through the shell, causing major structural changes in the pralines. For water- and liquor-based fillings, these changes may result in the cracking of the shell, reducing the shelf-life of the pralines [63]. It can be assumed that crack formation occurs either due to moisture or ethanol migration through the chocolate shell or due to an unbalanced distribution of moisture in the ganache filling, leading to the shrinking of some of the ganache’s parts and the expanding of others. As reported by Slettengren [63], both the water activity (*a_w_*) of the ganache filling and the praline geometry play an important role in the crack formation. The author observed that the pralines with an *a_w_* of 0.99 in the model filling cracked first and to the highest percentage, as compared to pralines with fillings with an *a_w_* of 0.86 and 0.78. For all the water activities in the model fillings, the round pralines began to crack first at a storage period of 28 days. For the ganache filling with an *a_w_* of 0.67, the square pralines did not crack at all, while the round pralines cracked at a low percentage. Based on these observations, it can be deduced that a square geometry may protect pralines against crack formation to a relatively high degree when combined with an optimized *a_w_* value of the ganache filling.

#### 4.1.2. Fat Bloom Development in Ganache-Filled Products

The occurrence of a white film on chocolate products, known as fat bloom, is a common issue in the confectionery industry, often leading to customer rejection of the product due to a decrease in visual quality [57]. The quality degradation is attributed to the migration of oil from a high-oil filling into the crystallized fat phase of the chocolate shell [64]. This phenomenon is particularly impactful in ganache-filled bonbons like pralines, especially when the center contains large quantities of highly mobile triacylglycerols (TAGs) that migrate through the shell to the surface. This oil migration is a triggering factor for cocoa butter (CB) re-crystallization, which leads to a greyer and duller appearance and can be accompanied by additional defects such as softening of the shell, hardening of the filling, and sensory deterioration [58,65]. The primary driving force behind the oil migration is typically a TAG concentration gradient between the fat in the liquid filling and the liquid cocoa butter in the chocolate shell, where the thermodynamic equilibrium is disturbed. The migrating liquid fat from the filling dissolves some of the crystallized CB TAGs in the shell, resulting in a softer chocolate shell, while the liquid CB from the shell’s fat matrix can migrate into the filling, leading to a harder texture upon re-crystallization [57].

Finding methods to reduce oil migration is a priority for the confectionery industry. The prevention of fat blooming would enable chocolate producers to better control the quality and extend the shelf-life of their products. To slow down fat migration, cocoa butter needs to be in a stable form with a low fraction of liquid fat. The β_V_ form is the preferred polymorph due to its valuable sensory attributes, stable microstructure, and ability to retard fat blooming [66,67,68,69]. During chocolate manufacturing, the most common method for obtaining a stable β_V_ form involves subjecting the chocolate to a specific temperature program under shear, i.e., a pre-crystallization process that induces the formation of a small proportion (1–3 vol.%) of the seed crystals, from which the remaining fat solidifies. The rate of oil migration in filled chocolates is directly proportional to the storage temperature. As reported by Depypere et al. [70] for milk chocolates with a hazelnut-based filling, storage at a low temperature (−20 °C or +4 °C) for part of the storage time reduced the appearance of visual fat blooming compared to the samples stored at +18 °C only, with no adverse effects on the products’ flavor. In particular, storage below 0 °C retarded the level of TAG migration, probably due to the microstructural (crystallization) changes. Another strategy to reduce fat migration may be to achieve a dense microstructure in the chocolate shell that prevents the liquid fat in the filling from reaching the product surface [58]. Another method is represented by the application of oleogel functional substances in the ganache filling, as described in Section 3.2.7.

The crystallization of the ganache system, and thereby the (un)development of fat blooming, seems to be primarily dependent on the composition and concentration of the substances used. Minor lipids such as mono- and diacylglycerols (MAGs, DAGs), cholesterol, and phospholipids could affect the crystallization of cocoa butter blends even at low concentrations, and they could thereby have a possible retarding effect on the formation of fat blooms [71]. Clercq et al. [65] investigated whether cocoa-butter-based diacylglycerols (CB DAGs) in filled chocolate could delay or prevent fat blooming. The authors observed the quality characteristics of dark chocolate containing up to 7.5% of CB DAGs on a fat basis over 1-year storage period. From the array of techniques employed (HPLC—high-performance liquid chromatography, DSC, texture analysis, automated image analysis), it was found that between 30 and 33 weeks of storage at 20 °C, a new phase of large fat crystals (β_VI_) occurred at the surface of the chocolate in contact with the filling. This was presumably a result of Ostwald ripening affected by the interactions between the cocoa butter and oil filling. This indicated that application of DAGs did not delay the oil migration, nor did it prevent the appearance of fat blooming; the profile of CB DAGs was probably like that of CB TAGs and was not able to induce significant changes. As noted by the authors, the oil migration induced fat polymorphic transformation (recrystallization) as a bulk phenomenon [65], which is consistent with the findings by Smith et al. [64], who reported that even a low level of nut oil in hazelnut-based filling may affect the fat transformation rate.

### 4.2. Shelf-Life Stability of Ganache Products

The shelf-life of ganache fillings is affected by several factors, including the quality of the raw materials and ingredients used (particularly the type of chocolate), the ratio of ingredients (especially the ratio of cream to chocolate), the product composition and structure, the moisture content, the water activity (*a_w_*), the pH, and the storage conditions [7,55]. Contamination from processing equipment and the environment may also play an important role [72]. The storage of handmade (filled) pralines can also present some shelf-life limitations due to the use of sensitive ingredients and due to changes during the processing and manipulation of the pralines, e.g., fat separation [26].

Several reasons for ganache’s susceptibility to microbial spoilage may be enumerated. The relatively high water content in ganache, along with substantial levels of sugars and proteins, provides an ideal growth medium for microorganisms tolerating a low *a_w_*, including xerophilic fungi, osmophilic yeasts [73], and pathogenic bacteria, causing slime, off flavors, and cracking of the product due to gas formation [62]. In particular, pathogenic bacteria such as *Salmonella* and *Listeria* spp. are the main relevant bacteria [22]. This microbial environment is a primary factor in reducing ganache’s shelf-life to max. 3–4 months. Moreover, the rich nutrient content (particularly fats) protects the microorganisms from conventional preservation treatments, allowing them to survive passage through the digestive tract by resisting stomach acidity [52]. Consequently, even a minimal microbial presence in such a matrix can lead to illness, as evidenced by some foodborne infections linked to *Salmonella*-contaminated chocolate [74]. This presents a significant challenge for ganache producers. Common strategies to prolong the shelf-life of ganache include the addition of thermally processed cream [26], lowering the water activity or pH levels, incorporating ethanol or preservatives [62], and achieving a balance between the ganache’s moisture and environmental humidity [22].

#### 4.2.1. Impact of Recipe Ingredients on Ganache Shelf-Life Stability

Izzreen et al. [1] reported that white chocolate ganaches can have a long shelf-life stability at a relevant total soluble solid (TSS) content. TSS is attributed to soluble substances consisting of sugars, hydrocolloids, and minerals. At a sugar content higher than 65 °Brix, the microbial growth in ganaches and chocolate products can be inhibited at intermediate levels of water activity (e.g., around 0.70–0.74) during storage at ambient (room) temperature [75].

As presented by Neuwirth et al. [7], specific food ingredients are able to enhance the microbial stability of ganache formulations, particularly if combined with a lower pH. The authors prepared a reference ganache from raspberry puree (32.23 wt.%), crystal sugar (4.87 wt.%), sorbitol (2.47 wt.%), chocolate (50.70 wt.%), and butter (4.78 wt.%). It was hypothesized that sorbitol, as a humectant, would stabilize the ganache moisture content while sugars (mono- and disaccharide) from the puree would contribute to an increase in osmotic pressure. The application of powdered milk (0.21 wt.%) and guar gum (1 wt.%) in the ganache formulation reduced the availability of water for the growth of microorganisms, as indicated by lower *a_w_* values. Overall, the studied samples of pralines (containing 60.60 wt.% of ganache filling) demonstrated a longer microbial stability after 4 weeks of incubation, with a CFU value under the limits issued by the International Standard Organization (ISO). This microbial stability was more favorable than the results presented by Van der Veken [76], who observed that ganache samples with a comparable water activity (greater than 0.85) had a shelf-life stability of less than two weeks.

#### 4.2.2. Effect of Preservation Techniques on Ganache Shelf-Life Stability

The shelf-life of ganache fillings can be extended employing suitable preservation methods applied to the product. The shelf-life stability of ganaches can significantly differ based on their composition. When stored at specific conditions (16 °C, protected from (sun)light), milk chocolate ganache containing sorbitol, invert sugar, and glucose syrup with a DE (dextrose equivalent) of 60 can last for 3 months and can be effectively applied to enrobed pralines during this time [77].

The shelf-life of the product can be modified depending on the time when the food is exposed to ambient air and moisture, with this being related to the storage temperature [78]. For ganache fillings, it is crucial to store the ganache in an airtight container (e.g., in a polypropylene box covered by polyethylene cling film [7], etc.) to prevent exposure to oxygen and moisture, which can cause discoloration and texture changes and accelerate microbial spoilage. Even with airtight storage being applied, ganache may still be vulnerable to bacterial and mold growth. Therefore, regular inspections for signs of spoilage, such as changes in odour, visible fungus formation between the filling and chocolate shell in pralines, and texture differences, are critical in ensuring the ganache remains fine to consume [22,79].

Refrigeration (at 4 ± 1 °C or lower temperatures) can preserve the quality of ganache products by reducing the microbiological growth of mesophilic organisms, slowing physiological processes (e.g., ripening), inhibiting biochemical reactions (e.g., lipid oxidation), and preventing physical changes (like desiccation). However, traditional refrigeration systems, particularly for long-term storage, are energy-consuming and have a notable environmental footprint. In contrast to this, high-pressure processing (HPP) is an emerging non-thermal technique that is able to inactivate microbes applying pressure without additives. Compared to standard refrigeration methods and heating treatments, HPP is characterized by a substantially reduced energy consumption with minimal heat loss (the adiabatic heating accounts for only ca. 3 °C/100 MPa) [37,52]. HPP as an effective alternative to conventional methods for extending the shelf-life of ganache fillings is discussed in Section 4.2.3.

#### 4.2.3. Impact of High-Pressure Processing on Ganache Shelf-Life Stability

Recent investigations have shown satisfactory results regarding the use of high-pressure processing (HPP) to extend the shelf-life of food products [80], accompanied by a higher preservation of natural aromas and fresh-like flavors [81]. For ganache fillings, the study conducted by Dias et al. [37] aimed to assess the impact of HPP (operated at 400 MPa for 2.5 min and at 500 MPa for 1 min) in relation to the storage temperature (4 °C and 20 °C) on the microbiological stability of filled chocolates over time. The research demonstrated that the stability of ganaches can be effectively influenced by both the HPP conditions and storage temperature. Microbiological parameters (CFU/g) were significantly affected in the HPP-treated samples; specifically, HPP at 400 MPa as well as at 500 MPa was shown to enhance the microbiological stability of the chocolate fillings, particularly in controlling mold and yeast proliferation, compared to the untreated samples stored at 20 °C (*p* < 0.05). A positive Pearson’s correlation was found between total aerobic mesophiles and storage time at 20 °C up to 60 days of storage, including in the samples treated with HPP at 400 MPa/20 °C and 500 MPa/20 °C. This correlation may be attributed to the survival capabilities of certain microorganisms post HPP treatment, particularly bacteria, which exhibit increased pressure resistance in nutrient-rich media (food emulsions) containing carbohydrates and proteins, such as ganache. Beyond 60 days, a decline in total aerobic mesophiles was observed across almost all the samples until 180 days. The only exception was the sample treated at 0.1 MPa/20 °C, potentially due to cracks in the chocolate couverture from moisture migration within the filling that occurred during the storage [60], which could lead to environmental contamination [37].

Panda et al. [52] analyzed dark chocolate fillings that were subjected to various pressure levels (400 and 600 MPa) and holding times (10 and 20 min) and then stored at a temperature of 20 °C for a period of 12 months. The HPP-treated samples were compared with unprocessed (control) batches stored at 4 °C and −12 °C. The products’ indigenous microflora was monitored at intervals of 2, 4, 6, 8, 10, and 12 months during the storage period. The authors observed that the 600 MPa/20 min processing appeared to be the most efficient for controlling mesophilic bacteria, exhibiting a count of 3.8 log CFU/g after a 12 month of storage time. Furthermore, this processing method proved effective in inactivating the presence of molds and yeasts in the chocolate fillings after the HPP treatment. Moreover, HPP at 600 MPa for 20 min was the only approach that fulfilled the recommended food safety standards after the storage period, although storage at a temperature of −12 °C (control batch) also provided lower mesophile counts. However, the fillings treated with these specific conditions showed a weaker structure compared to the control samples after 12 months of storage [52]. Overall, in terms of energy costs and efficiency during processing and storage, HPP may sustain a preferable industrial outcome over the vapour-compression refrigeration cycle.

## 5. Conclusions

This review addressed the current trends and challenges in the production and formulation of chocolate ganache, a confectionery blend of chocolate, sugars, and other ingredients. The emulsification process, a critical step that involves the dispersion of fat droplets from the chocolate and dairy components into a water-based phase, is instrumental in achieving the smooth and creamy texture that is characteristic of high-quality ganache. Its unique texture and flavor profile, which can be fine-tuned by adjusting the type of chocolate and proportions of ingredients used, make ganache a versatile component in a variety of culinary applications. In spite of the widespread use of ganache in the food industry, the scientific exploration of its properties remains relatively uncharted. This highlights the need for further research to fully understand the complex interactions between the various components in the ganache matrix.

The selection of the chocolate and other natural substances is a pivotal step in ganache profiling. Innovative approaches, such as the use of hydrocolloids, oleogels, and fruit- and other plant-based components, offer promising avenues for reducing the high caloric content of ganache fillings. Furthermore, the development of novel ganache variants through the exploration of different processing procedures and recipes opens exciting possibilities to avoid technological imperfections and to enhance the sensory attributes and shelf-life stability of ganache. In essence, a comprehensive understanding of the science behind ganache is crucial for optimizing its properties and, ultimately, for elevating the quality of ganache-filled products. This review underscores the importance of continued research in this area to advance the diversity of ganaches in the food industry. This knowledge will be instrumental in guiding future trends in ganache processing and recipe formulations. Innovative ganache-filled confections can yield substantial benefits for boutique chocolate producers, encompassing both small patisseries and large-scale chocolate factories. By diversifying the nutritional and flavor options for consumers, these new delightful ganaches can extend the reach of the chocolate market.

## Figures and Tables

**Table 1:** The calorie content and nutrient composition of chocolate ganache variants, as analyzed by Kim et al. [28].

Chocolate Ganache Variants	Energy (kcal)	Carbohydrate Content (wt.%)	Fat Content (wt.%)	Protein Content (wt.%)
Whipped cream ganache	436	25.5	35.5	4.0
Milk ganache	296	26.5	19.0	4.5
Vegetable cream ganache	386	25.5	30.0	4.1
Coconut milk ganache	359	25.4	26.7	4.0

**Table 2:** The nutrient composition of chocolate ganache with raspberry puree addition, as reported by Neuwirth et al. [7].

Ingredients	Content (wt.%)	Nutrients	Content (wt.%)
Raspberry puree	32.23	Total saccharides	36.70
Crystal sugar	4.87	Mono- and disaccharides	32.84
D-(+)-glucose	2.47	Total fats	22.48
D-(-)-fructose	2.47	Cocoa butter	18.56
Sorbitol	2.47	Milk fat	3.92
Chocolate	50.70	Saturated fatty acids	13.59
Butter	4.78	Total proteins	2.97
		Milk proteins	0.03
		Other proteins	2.94

**Table 3:** Formulations of white chocolate ganaches with different hydrocolloids applied at various concentrations compared to control samples (with no hydrocolloid), as investigated by Izzreen et al. [1] and Dias et al. [26].

	Carrageenan/Pectin/Xanthan Gum Content (wt.%) [1]					Iota-Carrageenan Content (wt.%) [26]		
Ingredients	Control	0.1	0.3	0.5	Ingredients	Control	0.5	1.0
White chocolate	49.0	49.0	49.0	49.0	White chocolate	22.0	22.0	22.0
Whipping cream	35.0	34.9	34.7	34.5	Skimmed milk	45.0	44.5	44.0
Unsalted cow butter	9.0	9.0	9.0	9.0	Sugar	22.0	22.0	22.0
Invert sugar	7.0	7.0	7.0	7.0	Invert sugar	11.0	11.0	11.0

**Table 4:** Formulations of ganache fillings with chilli flavor, as studied by Seçuk and Seçím [38].

Control Group Ganache (CG)		Powdered Chilli Pepper Ganache (PCP)		Chilli Pepper Seed Oil Ganache (CPS)	
Ingredients	Content (wt.%)	Ingredients	Content (wt.%)	Ingredients	Content (wt.%)
Cream	31.60	Cream	30.50	Cream	31.00
White chocolate	59.60	White chocolate	57.60	White chocolate	58.60
Butter	8.80	Butter	8.50	Butter	8.60
		Powder chilli pepper	3.40	Chilli pepper seed oil	1.70

**Table 5:** Formulation of ganache fillings with addition of Sacha inchi seeds, as investigated by Izzreen et al. [39].

Ingredients	Content (wt.%)	
	Ganache *with* Sacha Inchi	Ganache *without* Sacha Inchi (Control Sample)
White chocolate	48.0	48.0
Whipping cream	24.8	34.8
Cocoa butter	10.0	10.0
Invert sugar	7.0	7.0
Sacha inchi	10.0	-
Carrageenan	0.2	0.2

## Data Availability

No new data were created or analyzed in this study. Data sharing is not applicable to this article.

## References

[1] Izzreen I., Fisal A., Siti Norizah M.N. (2022). Effect of Hydrocolloids at Different Concentrations on the Physicochemical Properties and Particle Size Distribution of White Chocolate Ganache. Malays. Cocoa J..

[2] McGill J., Hartel R.W. (2018). Investigation into the Microstructure, Texture and Rheological Properties of Chocolate Ganache. J. Food Sci..

[3] Hermé P., Martiniere B.L. (2018). Macarons.

[4] Palczak J., Giboreau A., Rogeaux M., Delarue J. (2020). How do Pastry and Culinary Chefs Design Sensory Complexity?. Int. J. Gastron. Food Sci..

[5] Rigg A. (2014). Macarons: Chic and Delicious French Treats.

[6] McGill J., Hartel R., Hofberger R. (2018). The Art and Science of Ganache. Manuf. Confect..

[7] Neuwirth V., Lapčíková B., Lapčík L., Valenta T., Míšková Z. (2024). Effect of Technological Processing and Recipe Formulation on the Physico-Chemical Properties of Ganaches and Chocolate Pralines. J. Food Eng..

[8] Merachli F., Devienne J., Delmas R., Plawinski L., Leal-Calderon F., Delample M. (2021). Impact of Cocoa Fibers on the Stability and Rheological Properties of Chocolate Ganaches. LWT.

[9] Goralchuk A., Gubsky S., Omel’chenko S., Riabets O., Grinchenko O., Fedak N., Kotlyar O., Cheremska T., Skrynnik V. (2020). Impact of Added Food Ingredients on Foaming and Texture of the Whipped Toppings: A Chemometric Analysis. Eur. Food Res. Technol..

[10] Park M.A., Lee K.J., Kim S.J., Kim M.R. (2020). Quality Characteristics and Antioxidant Activities of Ganache Added with *Porphyra Tenera* Powder. Korean J. Food Preserv..

[11] Cubides Y.T.P. (2014). Developing Milk Protein Based Structure for New Dairy Products.

[12] Yilmaz İ., Doğan G. (2023). Product Development and Sensory Evaluation of Dark Chocolate Filled with Chestnut Honey. Black Sea J. Agric..

[13] Popov-Raljić J.V., Laličić-Petronijević J.G., Georgijev A.S., Popov V.S., Mladenović M.A. (2010). Sensory Evaluation of Pralines Containing Different Honey Products. Sensors.

[14] Clark C., Miller J.P., Van Buiten C. (2022). Chocolate. Superfoods. Cultural and Scientific Perspectives.

[15] Norton J.E., Fryer P.J. (2012). Investigation of Changes in Formulation and Processing Parameters on the Physical Properties of Cocoa Butter Emulsions. J. Food Eng..

[16] Norton J.E., Fryer P.J., Parkinson J., Cox P.W. (2009). Development and Characterisation of Tempered Cocoa Butter Emulsions Containing Up to 60% Water. J. Food Eng..

[17] Greweling P.P. (2012). Chocolates and Confections: Formula, Theory, and Technique for the Artisan Confectioner.

[18] Saglio A., Bourgeay J., Socrate R., Canette A., Cuvelier G. (2018). Understanding the Structure of Ganache: Link between Composition and Texture. Int. J. Gastron. Food Sci..

[19] Kim S.M., Woo J.H., Kim H.W., Park H.J. (2022). Formulation and Evaluation of Cold-Extruded Chocolate Ganache for Three-Dimensional Food Printing. J. Food Eng..

[20] Herrero D., Etienne G. (2009). Pâtisserie, Les Clés De La Réussite—Coffret En 2 Volumes.

[21] Leal-Calderon F., Thivilliers F., Schmitt V. (2007). Structured Emulsions. Curr. Opin. Colloid Interface Sci..

[22] Wybauw J. (2010). Fine Chocolates, Great Experience 3: Extending Shelf Life.

[23] Greweling P. (2007). The Crystallization of Ganache. Manuf. Confect..

[24] Peyronel F., Pink D.A. (2021). Using USAXS to Predict the Under-Tempered Chocolate Microstructure. Food Res. Int..

[25] Dias J., Alvarenga N., Sousa I. (2015). Effect of Hydrocolloids on Low-Fat Chocolate Fillings. J. Food Sci. Technol..

[26] Dias J., Alvarenga N., Sousa I. (2017). Shelf-Life of Reduced-Fat White Chocolate Fillings using Iota-Carrageenan. Emir. J. Food Agric..

[27] Indiarto R., Situmorang A.K.N., Harunaningtyas A., Arifin H.R., Subroto E., Herawati E.R.N., Djali M., Mahani, Muhammad D.R.A. (2024). Reformulation of White Chocolate with Soy- and Coconut-Based Vegetable Ingredients Incorporating Encapsulated Cinnamon Extract: Investigation of Physicochemical, Antioxidant, and Sensory Properties. Int. J. Food Prop..

[28] Kim Y.J., Kang S., Kim D.H., Kim Y.J., Kim W.R., Kim Y.M., Park S. (2017). Calorie Reduction of Chocolate Ganache through Substitution of Whipped Cream. J. Ethn. Foods.

[29] Gonçalves E.V., Caetano Da S., Lannes S. (2010). Chocolate Rheology. Ciênc. Tecnol. Aliment..

[30] Glicerina V., Balestra F., Rosa M.D., Romani S. (2014). Microstructural and Rheological Properties of White Chocolate during Processing. Food Bioprocess Technol..

[31] Kumbár V., Kouřilová V., Dufková R., Votava J., Hřivna L. (2021). Rheological and Pipe Flow Properties of Chocolate Masses at Different Temperatures. Foods.

[32] Cavella S., Miele N.A., Fidaleo M., Borriello A., Masi P. (2020). Evolution of Particle Size Distribution, Flow Behaviour and Stability during Mill Ball Refining of a White Chocolate Flavouring Paste. LWT.

[33] Neuwirth V. (2023). Effect of the Processing and Composition of Chocolate Pralines’ Fillings on their Final Quality. Master’s Thesis.

[34] Costa C., Medronho B., Filipe A., Mira I., Lindman B., Edlund H., Norgren M. (2019). Emulsion Formation and Stabilization by Biomolecules: The Leading Role of Cellulose. Polymers.

[35] Galanakis C.M. (2021). Food Structure and Functionality.

[36] Bai L., Liu F., Xu X., Huan S., Gu J., McClements D.J. (2017). Impact of Polysaccharide Molecular Characteristics on Viscosity Enhancement and Depletion Flocculation. J. Food Eng..

[37] Dias J., Coelho P., Alvarenga N.B., Duarte R.V., Saraiva J.A. (2018). Evaluation of the Impact of High Pressure on the Storage of Filled Traditional Chocolates. Innov. Food Sci. Emerg. Technol..

[38] Seçuk B., Seçím Y. (2022). Development of Chili Pepper Ganache Filled Chocolate in Artisan Chocolate Production, Determination of Sensory and Physicochemical Characteristics. Food Sci. Technol..

[39] Izzreen I., Ly S.K., Khaironi J., Fisal A., Seng N.S.S., Ghani M.A. (2023). Physicochemical, Total Phenolic Content, Antioxidant Activity, and Sensory Acceptability of Milk and Dark Chocolates Filled with Sacha Inchi Ganache. Malays. Cocoa J..

[40] Beal K. (2019). Considerations in the Addition of Cannabis to Chocolate. Curr. Opin. Food Sci..

[41] Rupasinghe H.P.V., Davis A., Kumar S.K., Murray B., Zheljazkov V.D. (2020). Industrial Hemp (*Cannabis sativa* Subsp. *Sativa*) as an Emerging Source for Value-Added Functional Food Ingredients and Nutraceuticals. Molecules.

[42] Steinbach W. (1999). Hemp. Pralines. Patent.

[43] Bartončíková M., Lapčíková B., Lapčík L., Valenta T. (2023). Hemp-Derived CBD used in Food and Food Supplements. Molecules.

[44] Scharfe M., Flöter E. (2020). Oleogelation: From Scientific Feasibility to Applicability in Food Products. Eur. J. Lipid Sci. Technol..

[45] Bot A., Flöter E., Marangoni A.G., Garti N. (2018). Chapter 2—Edible Oil Oleogels Based on Self-assembled β-Sitosterol+γ-Oryzanol Tubules. Edible Oleogels.

[46] Sun P., Xia B., Ni Z., Wang Y., Elam E., Thakur K., Ma Y., Wei Z. (2021). Characterization of Functional Chocolate Formulated using Oleogels Derived from Β-Sitosterol with Γ-Oryzanol/Lecithin/Stearic Acid. Food Chem..

[47] Matheson A.B., Koutsos V., Dalkas G., Euston S., Clegg P. (2017). Microstructure of Β-Sitosterol:Γ-Oryzanol Edible Organogels. Langmuir.

[48] Pinto T.C., Martins A.J., Pastrana L., Pereira M.C., Cerqueira M.A. (2022). Water-in-Oleogel Emulsion Based on Γ-Oryzanol and Phytosterol Mixtures: Challenges and its Potential use for the Delivery of Bioactives. J. Am. Oil Chem. Soc..

[49] Wendt A., Abraham K., Wernecke C., Pfeiffer J., Flöter E. (2017). Application of Β-Sitosterol + Γ-Oryzanol-Structured Organogel as Migration Barrier in Filled Chocolate Products. J. Am. Oil Chem. Soc..

[50] Fernandes Almeida R., Aguiar Borges L., Torres da Silva T., Serafim Timóteo dos Santos N., Gianasi F., Augusto Caldas Batista E., Efraim P. (2024). Chocolates, Compounds and Spreads: A Review on the use of Oleogels, Hydrogels and Hybrid Gels to Reduce Saturated Fat Content. Food Res. Int..

[51] Balasubramaniam V.M., Farkas D., Turek E.J. (2008). Preserving Foods through High-Pressure Processing. Food Technol. Mag..

[52] Panda A., Coelho P., Alvarenga N.B., Silva J.L., Lampreia C., Santos M.T., Pinto C.A., Amaral R.A., Saraiva J.A., Dias J. (2022). Effect of High Pressure on the Properties of Chocolate Fillings during Long-Term Storage. Foods.

[53] Nopens I., Foubert I., De Graef V., Van Laere D., Dewettinck K., Vanrolleghem P. (2008). Automated Image Analysis Tool for Migration Fat Bloom Evaluation of Chocolate Coated Food Products. LWT.

[54] Briones V., Aguilera J.M., Brown C. (2006). Effect of Surface Topography on Color and Gloss of Chocolate Samples. J. Food Eng..

[55] Subramaniam P.J., Talbot G. (2009). Shelf-life prediction and testing. Science and Technology of Enrobed and Filled Chocolate, Confectionery and Bakery Products.

[56] Tan T.Y.C., Lim X.Y., Yeo J.H.H., Lee S.W.H., Lai N.M. (2021). The Health Effects of Chocolate and Cocoa: A Systematic Review. Nutrients.

[57] Dahlenborg H., Millqvist-Fureby A., Bergenståhl B. (2015). Effect of Shell Microstructure on Oil Migration and Fat Bloom Development in Model Pralines. Food Struct..

[58] Svanberg L., Ahrné L., Lorén N., Windhab E. (2011). Effect of Pre-Crystallization Process and Solid Particle Addition on Cocoa Butter Crystallization and Resulting Microstructure in Chocolate Model Systems. Procedia Food Sci..

[59] Yu D., Xue Z., Mu T. (2021). Eutectics: Formation, Properties, and Applications. Chem. Soc. Rev..

[60] Svanberg L., Lorén N., Ahrné L. (2012). Chocolate Swelling during Storage Caused by Fat or Moisture Migration. J. Food Sci..

[61] Popov-Raljić J.V., Laličić-Petronijević J.G. (2009). Sensory Properties and Color Measurements of Dietary Chocolates with Different Compositions during Storage for Up to 360 Days. Sensors.

[62] Marvig C.L., Kristiansen R.M., Madsen M.G., Nielsen D.S. (2014). Identification and Characterisation of Organisms Associated with Chocolate Pralines and Sugar Syrups used for their Production. Int. J. Food Microbiol..

[63] Slettengren K. (2010). Crack Formation in Chocolate Pralines. Master’s Thesis.

[64] Smith K.W., Cain F.W., Talbot G. (2007). Effect of Nut Oil Migration on Polymorphic Transformation in a Model System. Food Chem..

[65] Clercq N.D., Depypere F., Delbaere C., Nopens I., Bernaert H., Dewettinck K. (2014). Influence of Cocoa Butter Diacylglycerols on Migration Induced Fat Bloom in Filled Chocolates. Eur. J. Lipid Sci. Technol..

[66] Kinta Y., Hatta T. (2005). Composition and Structure of Fat Bloom in Untempered Chocolate. J. Food Sci..

[67] Kinta Y., Hatta T. (2007). Composition, Structure, and Color of Fat Bloom due to the Partial Liquefaction of Fat in Dark Chocolate. J. Am. Oil Chem. Soc..

[68] Timms R.E. (2002). Oil and Fat Interactions. Manuf. Confect..

[69] Ziegler G.R., Shetty A., Anantheswaran R.C. (2004). Nut Oil Migration through Chocolate. Manuf. Confect..

[70] Depypere F., De Clercq N., Segers M., Lewille B., Dewettinck K. (2009). Triacylglycerol Migration and Bloom in Filled Chocolates: Effects of Low-Temperature Storage. Eur. J. Lipid Sci. Technol..

[71] Tietz R.A., Hartel R.W. (2000). Effects of Minor Lipids on Crystallization of Milk Fat-Cocoa Butter Blends and Bloom Formation in Chocolate. J. Am. Oil Chem. Soc..

[72] Palomino Camargo C.E., Sira E.E.P. (2015). Chapter II. Microbiological and Physicochemical Factors that Affect the Safety and Quality of Chocolate. Chocolate: Cocoa Byproducts Technology, Rheology, Styling, and Nutrition.

[73] De Clercq N., Van Coillie E., Van Pamel E., De Meulenaer B., Devlieghere F., Vlaemynck G. (2015). Detection and Identification of Xerophilic Fungi in Belgian Chocolate Confectionery Factories. Food Microbiol..

[74] do Nascimento M.d.S., Brum D.M., Pena P.O., Berto M.I., Efraim P. (2012). Inactivation of *Salmonella* during Cocoa Roasting and Chocolate Conching. Int. J. Food Microbiol..

[75] Miquelim J.N., Alcântara M.R., Lannes S.C.d.S. (2011). Stability of Fruit Bases and Chocolate Fillings. Food Sci. Technol..

[76] Van der Veken E. (2019). Balancing Technological Sugars in Ganaches: Introduction to the Use of Technological Sugars in Ganaches.

[77] (2024). Milk Chocolate Ganache for Moulded Pralines. https://www.callebaut.com/en/chocolate-recipe/1421/milk-chocolate-ganache-moulded-pralines.

[78] Alessandro Del Nobile M., Conte A. (2023). Secondary Shelf Life of Foods: State of the Art and Future Perspective. Food Eng. Rev..

[79] (2024). Is Ganache Shelf Stable? The Importance of Chocolate Shelf Life.

[80] Nabi B.G., Mukhtar K., Arshad R.N., Radicetti E., Tedeschi P., Shahbaz M.U., Walayat N., Nawaz A., Inam-Ur-Raheem M., Aadil R.M. (2021). High-Pressure Processing for Sustainable Food Supply. Sustainability.

[81] Sehrawat R., Kaur B.P., Nema P.K., Tewari S., Kumar L. (2021). Microbial Inactivation by High Pressure Processing: Principle, Mechanism and Factors Responsible. Food Sci. Biotechnol..

